# The relationship between sleep disorder and mental health in athletes and its mediating role: a cross-sectional study

**DOI:** 10.1371/journal.pone.0319813

**Published:** 2025-03-24

**Authors:** Zejun Yan, Lei Wang

**Affiliations:** 1 Shanghai Nanhui Middle School, Shanghai, China; 2 Shanghai University of Sport, Shanghai, China; Portugal Football School, Portuguese Football Federation, PORTUGAL

## Abstract

The mental health of high-level athletes is a growing concern, especially in the context of the COVID-19 pandemic. Sleep is also an explicit health index closely related to mental health. This study aims to investigate the relationship between sleep, anxiety, depression, and perceived psychological stress among athletes, with a focus on sleep as a potential mediator in these mental health conditions. A comprehensive questionnaire included the Generalized Anxiety Disorder Scale-7 (GAD-7), Patient Health Questionnaire-9 (PHQ-9), Athlete Psychological Stress Questionnaire (APSQ), and Athlete Sleep Screening Questionnaire (ASSQ), and was administered to a sample of 501 elite Chinese athletes. Statistical analysis software and structural equation modeling were used to examine the characteristics, correlations, and influence pathways of the four indicators. The findings indicated that, compared to previous studies, the prevalence of perceived psychological stress (78.6%) and sleep disorders (24.8%) in the athlete sample were higher. All three mental health indicators were significantly positively correlated with sleep disorders. In terms of influence pathways, there was a direct path from APSQ to PHQ-9, with the direct effect of perceived stress on depression accounting for 32.5%. The indirect paths from APSQ to GAD-7 to PHQ-9 and from APSQ to GAD-7 to ASSQ to PHQ-9 accounted for 67.5%. Sleep disorders in competitive athletes are closely related to mental health, potentially serving as an important observable health behavior indicator and mediating factor in the transition from anxiety to depression. There is a need to strengthen comprehensive intervention measures that combine mental health and sleep health behaviors for athletes.

## Introduction

China’s competitive sports have achieved remarkable success and delivered impressive performances on the international stage. Statistics from international sports organizations indicate that Chinese athletes have demonstrated their strength and influence in competitive sports by winning medals in major international events, including the Olympics, World Cups, and World Championships [1]. In addition to their outstanding competitive abilities, the mental health of athletes has also received significant attention. Under the significant training load and competitive pressure, athletes are susceptible to psychological issues such as anxiety and depression. This concern has intensified, particularly in the wake of the COVID-19 pandemic, which has further heightened the perceived psychological stress experienced by athletes [2]. As a result, this phenomenon has attracted increased attention within the field of sports research. Research has shown that mental health is closely linked to the competitive performance of athletes [3]. Therefore, addressing the mental health issues faced by high-level athletes is essential. This includes the development of mental health education programs, the provision of psychological counseling services, and the implementation of effective coping strategies to support athletes in managing their daily training and competition. In March 2019, Mental health in elite athletes: *International Olympic Committee Consensus Statement* was published, which proposed a standardized, evidence-based measure of mental health symptoms in elite athletes [4]. That same year, Consensus statement on improving the mental health of high-level athletes was also released by the International Society of Sport Psychology [5], advocating for a definition of sport-specific mental health and the creation of a comprehensive mental health assessment strategy tailored to the context of sports.

Athlete Psychological Strain encompasses the various psychological disturbances and challenges that athletes perceive they encounter during sports competitions. This perception of stress is influenced by the unique characteristics of competitive sports, including the intense competition, significant responsibilities, and high expectations that athletes perceive [6]. While appropriate levels of psychological stress can enhance competitive performance [7], prolonged exposure to high-intensity stress can adversely affect both physical and mental health [8]. Tools for measuring psychological stress in athletes include the Brief Psychological Status Rating Scale (Kessler-10) [9]and the Athlete Psychological Strain Questionnaire (APSQ) [10], among others. The perception of psychological stress can lead to mental health symptoms, including anxiety, depression, and mental fatigue. Extended psychological stress may impair athletes’ ability to fulfill daily training and competition tasks, potentially leading to their withdrawal from the sport.

Among these symptoms, anxiety is the most frequently reported mental health issue among athletes. Anxiety is defined as a physical and psychological response that arises when an individual faces a perceived threat or stress. It can manifest through various symptoms, including nervousness, restlessness, fear, rapid heartbeat, shortness of breath, muscle tension, headaches, insomnia, fatigue, and difficulty concentrating [11]. Current tools for assessing anxiety include the Zung’s Self-rating Anxiety Scale (SAS) [12]and the Generalized Anxiety Disorder Scale-7 Item (GAD-7) [13]. Factors influencing anxiety can be categorized into individual, environmental [14], sport-related [15], and life-related domains [16].

Depression is a more severe mental health concern compared to anxiety, characterized by mood changes, a loss of interest or pleasure in daily activities, and associated symptoms such as sleep and appetite change, decreased energy, lack of concentration, and thoughts of death or suicidal attempts. Studies indicate that one-third of high-level athletes exhibit depressive symptoms, with depression being more prevalent among this group than in the general population [17]. For instance, the detection rate of major depression among high-level athletes in Germany is as high as 28.6% [18]. Particularly in the context of the COVID-19 pandemic, elite athletes facing postponed or cancelled competition schedules and alterations in training methods [19] and other research have reported increased levels of depression [20]. Thus, high-level athletes may represent a demographic that is more mentally burdened and challenged by the impacts of COVID-19 pandemic [21].

Sleep is a crucial recovery mechanism for athletes, essential for optimal performance and overall health [22]. It is recognized as the primary and most effective strategy for athletes to manage stress [23]. However, elite athletes often encounter sleep disturbances due to factors such as early morning training, stress and jet lag [24,25,26]. A study involving Swiss Olympic participants revealed that 20-34% of elite athletes experienced sleep issues during the Olympics. Furthermore, research indicated that 49% of high-level athletes in Australia reported insufficient actual hours of sleep [27]. Additionally, studies have established a strong correlation between sleep and the mental health of athletes. For instance, sleep influences subjective well-being [28], affects the severity of depressive symptoms [29,30,31], and predicts fluctuations in symptoms of depression and anxiety [32].

In summary, there is a strong relationship between mental health and sleep. However, limited research has been conducted with high-level athlete populations, particularly regarding the role of sleep in conjunction with various mental health monitoring tools. Adequate sleep behavior and mental health status are essential for facilitating the participation of high-level athletes in training. Both sleep and mental health have been recognized as critical factors in the management and rehabilitation of these athletes [33]. Thus, there is a pressing need to examine the interplay between mental health and sleep within this population. The primary aim of this study was to investigate the relationship between sleep and anxiety, depression, and perceived psychological stress among athletes, while also assessing the mediating role of sleep between the three mental health monitoring tools. The study posited that sleep is significantly correlated with anxiety, depression, and perceived psychological stress, and that it partially mediates the relationships between perceived psychological stress and anxiety, perceived psychological stress and depression, as well as anxiety and depression.

## Materials and methods

### Design and participants

The target population for this study is active athletes who are at least 18 years old with grades ranging from Division I Athlete, Master Athlete, to International Master Athlete, based on the standards set by the Chinese National Sports Administration [34].An online questionnaire was created for this research using the web-based tool “Sojump” (WJX.co.uk). Before participating in the study, athletes were provided with information about the research, and their written informed consent was obtained. Data collection was conducted anonymously. The exclusion criteria for participants in the questionnaire study included: (1) individuals younger than 18 or older than 30 years; (2) those who were retired at the time of the survey; (3) athletes below the highest competitive level; (4) instances of duplicate or suspiciously consistent responses; and (5) completion times for the questionnaires shorter than 300 seconds.

### Procedures

#### This study received approval from the ethics review committee of the Shanghai.

University of Sport (Approval No.:102772022RT113). The information about the study was disseminated through social media and sent to key stakeholders, including the Athletics Administration Center, Wushu Administrative Center, Water Sports Administrative Center, Table Tennis and Badminton Administrative Center, Gymnastics Administrative Center, Shooting and Archery Administrative Center, and Weighting and Combat Sports Administrative Center of the General Administration of Sport of China, as well as the Guangdong Provincial Sports Bureau. These stakeholders were requested to share the information with their respective athletes. Additionally, college athletes from the authors’ organizations were recruited through open calls, and they were encouraged to inform anyone they knew who met the inclusion criteria. The data was collected from January 31 to March 31, 2023. Prior to the online survey, athletes were required to sign an informed consent and they were required to confirm that their participation would remain confidential, that they could withdraw from the study after completing the survey, that they were not obligated to answer any questions they preferred to skip, and that only the research team would have access to their information. Each athlete received a personalized link upon completion of the survey, allowing them to access the results of the relevant scale. After the final data screening, a total of 501 data were validated. Among these, 413 (56.6%) were Division I athletes, 269 (36.8%) were Master Athletes, and 48 (6.6%) were International Master Athletes. These participants were involved in 22 different sports, with the top five being athletics (21.8%), diving (14.7%), swimming (12.2%), skills (9.9%), and tennis (8.9%). Further specific information is provided in the attached table.

### Measures

In this study, a comprehensive survey was conducted using a passage scale appropriate for the athlete population. The questionnaire primarily comprises several components. The first component gathers basic information from the survey respondents, including gender, age, athlete level, sports specialty, and other relevant details. Following this, the questionnaire addresses scales related to anxiety, depression, psychological stress, and sleep.

The validation criteria for the reliability of the scale stipulate that the correlation between each indicator and the total score of the scale must exceed 0.4 [35]. The fitting procedure involved randomly dividing the sample into two equal datasets. An exploratory factor analysis was conducted on the first dataset to determine whether the results of the main factor extraction aligned with the original design of the scale [36]. If the structure of the principal factors differed from the original design, the dimensions were redefined based on the principal factor extraction [37]. Validation of the structural equation modeling was performed using the second dataset, ensuring that the fit met the appropriate criteria [Kline] to finalize the newly revised scale structure. The content reliability of the scale was quantified using Cronbach’s coefficient [38].

Psychological stress was assessed using the Athlete Psychological Strain Questionnaire (APSQ), which comprises 10 questions, such as “It was difficult to be around teammates,” “I found it difficult to do what I needed to do,” and “ I was less motivated.” Responses were scored on a 5-point Likert scale, with each item receiving a score ranging from 1 to 5 points, resulting in a total score between 10 and 50 points. A score of 15 or less indicates no stress, scores between 15 and 16 indicate moderate stress, scores from 17 to 19 indicate high stress, and scores of 20 or above indicate very high stress, with scores of 17 or higher necessitating clinical observation. The APSQ consists of three dimensions: Self-Control Difficulties (items 1-4), Performance Anxiety (items 5-8), and External Coping (items 9-10). Previous studies have demonstrated that the scale possesses high internal consistency [39]. In this study, items 1 and 10 were excluded following correlation analysis and exploratory factor analysis. The results of the exploratory factor analysis revealed that the factor loadings of the 1 item and 10 item in were below 0.4, which did not show the correlation between the variables and the observed indicators well, so we finally decided to use questions 2-9 item. The adjusted scale yielded two dimensions: factor 1, ‘Self-Control ‘ (original items 2-5), and factor 2, ‘Future Stress Coping’ (original items 6-9). Structural equation modeling confirmed the satisfactory validity of the new scale structure, and the internal consistency of the adapted scale was found to be fair (α =  0.756).

Anxiety was assessed using the Generalized Anxiety Disorder (GAD-7) scale, which comprises seven questions, (e.g., “How often have you been bothered by feeling nervous, anxious, or on edge in the past 2weeks?”), Participants rate their experiences on a 5-point Likert scale, ranging from 0 (“not at all”) to 3 (“nearly every day”). The total score can range from 0 to 21, with scores of 5 or less indicating no anxiety, scores of 5-9 indicating mild anxiety, scores of 10-14 indicating moderate anxiety, scores of 15 and above indicating severe anxiety, and scores of ≥ 10 necessitating clinical observation. The GAD-7 has undergone rigorous reliability validation [40]. The single-dimensional structure of the original scale was preserved following correlation and exploratory factor analyses, with favorable results from the validated factor analysis utilizing structural equation modeling. The internal consistency of the scale was found to be satisfactory (α =  0.920).

The depression scale was derived from the Patient Health Questionnaire (PHQ-9), which consists of nine questions, such as “Over the last two weeks, how often have you been bothered by the following problems, little interest or pleasure in doing things?” This scale also employs a 5-point Likert scale, ranging from 0 (“not at all”) to 3 (“nearly every day”). Total scores range from 0 to 27, with scores of 5 or less indicating no depression, scores of 5 to 9 indicating mild depression, scores of 10 to 14 indicating moderate depression, scores of 15-19 indicating moderately severe depression, and scores of 20 and above indicating severe depression. Typically, scores of ≥ 10 require clinical observation. The PHQ-9 has been extensively validated for reliability, demonstrating strong reliability in previous studies [41]. Correlation and exploratory factor analyses were conducted to ensure the retention of the single-dimensional structure of the original scale. The internal consistency of the scale was confirmed to be good after data fitting (Cronbach’s coefficient *α* =  0.896).

The Athlete Sleep Screening Questionnaire (ASSQ) is a tool designed to assess sleep patterns among athletes. It comprises nine questions that inquire about various aspects of sleep, including the number of hours of actual sleep get at night in recent times, the level of satisfaction with sleep quality, and the duration required to fall asleep each night, etc. The total score is calculated by summing the scores of items 1, 3, 4, 5, and 6, resulting in a range from 0 to 17. Scores are categorized as follows: 0 to 4 indicates no sleep disorder, 5 to 7 indicates a mild sleep disorder, 8 to 10 indicates a moderate sleep disorder, and 11 to 17 indicates a severe sleep disorder. Previous studies has shown strong internal consistency and reliability in previous studies. It is currently the most widely utilized sleep screening tool within the athlete population [42]. Correlation and exploratory factor analyses have confirmed its good internal consistency, while preserving the original scale’s single-dimensional structure (Cronbach’s coefficient *α* =  0.751).

### Data analysis

The study utilized IBM SPSS v26 software for descriptive statistics, correlation analysis between variables, exploratory factor analysis, and structural equation analysis, employing Amos 23.0 software to validate the fitness of the scales and to assess the influence of the ASSQ as a mediating variable. Maximum likelihood estimation was applied to calculate the estimates. Following Hooper’s recommendations [43], the goodness-of-fit criteria for each structural equation model were established as follows: comparative fit index (CFI) >  0.9, Tucker-Lewis Index (TLI) >  0.9, root mean square error of approximation (RMSEA) <  0.08, and a ratio of 1 ≤  X2/df ≤  3, which were deemed acceptable. For the analyses conducted in Amos, bias-corrected bootstrap methods were employed to test for indirect effects, utilizing 2000 bootstrap samples. Confidence intervals were computed at the 95% level, and missing data points were estimated using the sample mean.

## Results

Table 1 presents the mean, standard deviation, skewness, and kurtosis for the total scores, dimensions, and individual question items across the four indicators. Notably, item 9 of the PHQ-9 and item 6 of the ASSQ exhibited suboptimal performance regarding skewness and kurtosis, whereas the other scale question items demonstrated satisfactory performance. Following a thorough evaluation, the decision was made to retain these items for the purposes of this study.

**Table 1:** Descriptive statistics.

	Mean	Standard Deviation	Skewness	Kurtosis
GAD-7	5.03	4.326	1.094	1.597
Item-1	1.90	0.783	0.902	0.898
Item-2	1.74	0.787	1.082	1.075
Item-3	1.75	0.780	1.052	1.057
Item-4	1.85	0.811	0.987	0.847
Item-5	1.63	0.765	1.307	1.674
Item-6	1.59	0.731	1.308	1.778
Item-7	1.57	0.728	1.337	1.800
PHQ-9	5.81	4.652	1.192	2.360
Item-1	1.74	0.743	1.013	1.226
Item-2	1.71	0.739	1.056	1.299
Item-3	1.80	0.816	1.010	0.761
Item-4	2.07	0.812	0.708	0.314
Item-5	1.67	0.752	1.139	1.289
Item-6	1.56	0.737	1.319	1.554
Item-7	1.60	0.737	1.258	1.587
Item-8	1.44	0.659	1.651	3.012
Item-9	1.23	0.568	2.996	9.731
APSQ-8	18.28	5.505	0.248	-0.187
Dimension-1	9.32	3.208	0.331	-0.167
Dimension-2	8.97	2.907	0.388	0.001
ASSQ	5.94	2.833	0.850	0.769
item-1	2.61	0.893	0.125	-0.167
item-3	2.66	1.057	0.506	-0.470
item-4	1.85	0.860	0.823	0.028
item-5	1.94	0.811	0.678	0.103
item-6	1.11	0.473	4.672	22.257

According to the criteria established by Evans (1996), there is a weak correlation between the ASSQ and GAD-7, while a moderate correlation is observed between the ASSQ and PHQ-9. Additionally, a strong correlation exists between two of the three mental health indicators: GAD-7, PHQ-9, and APSQ (Table 2).

**Table 2:** Relationship between mental health and sleep (Note: Gender and sport level as moderating variables, ***p < 0.001).

	1	2	3	4	5	6
1. GAD-7	1					
2. PHQ-9	0.794***	1				
3. APSQ-8	0.656***	0.658***	1			
4. --Dimension-1	0.617***	0.616***	0.914***	1		
5. --Dimension-2	0.569***	0.575***	0.897***	0.641***	1	
6. ASSQ	0.369***	0.463***	0.335***	0.300***	0.307***	1

**Fig 1:**
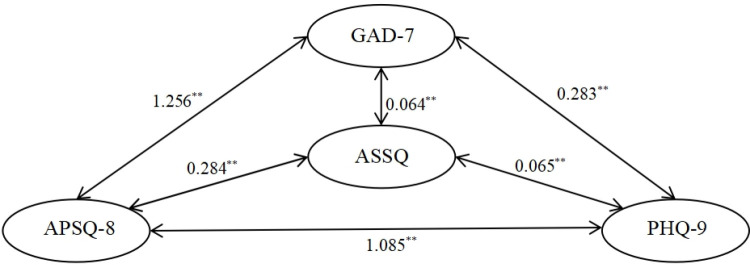
Model for the path analysis between anxiety, depression, psychological stress and sleep. (Note. X2 =  560.746, df =  202, X2/df =  2.776, p =  0.000, SRMR =  0.0483, RMSEA =  0.060, CFI =  0.940, GFI =  0.914).

To investigate the pathways of influence among the three mental health indicators (APSQ, GAD-7, PHQ-9) and sleep disturbance (ASSQ), the structural validity and reliability were assessed based on 23 observed variables: 7 items of GAD, 9 items of PHQ, 2 sub-dimensions of APSQ, and 5 items of ASSQ. Four latent variables were analyzed: anxiety, depression, psychological stress, and sleep disturbance. The confirmatory factor analysis (CFA), utilizing the maximum likelihood (ML) method, indicated a good fit to the data, with the following results: X² =  560.7, df =  202, X²/df =  2.78, p =  0.000, TLI =  0.925, GFI =  0.914, CFI =  0.940, RMSEA =  0.060, and SRMR =  0.0483. The relationships between the internal sub-dimensions were fine-tuned accordingly [44]. The final results of the data fit for this structural equation model were satisfactory, exhibiting significant standardized regression coefficients across paths, as illustrated in Fig 1.

As shown in Table 3, the structural reliability values (CR) of the indicators in the model ranged from 0.70 to 0.91, with all values exceeding 0.70, thereby satisfying the established requirements [36]. This indicates that all latent variables exhibited good structural reliability. In terms of average variance extracted (AVE), the AVE values for PHQ-9 and ASSQ were below 0.50, while the remaining two variables exceeded 0.50. The convergent validity of the latent variables was assessed to be between good and fair, and both the convergent and discriminant validity of the model’s variables met the necessary criteria.

**Table 3:** Model reliability and squared deviation extraction (Note: Φ denotes the root value using the correlation coefficient).

Norm	Structural Reliability	Extracted Mean Square Error	Inter-indicator correlation coefficient (Φ)		
GAD-7	PHQ-9	APSQ
GAD-7	0.9079	0.5861	1		
PHQ-9	0.8690	0.4276	0.794 (0.8911)	1	
APSQ-8	0.7673	0.6229	0.658 (0.8112)	0.660 (0.8124)	1
ASSQ	0.7099	0.3751	0.372 (0.6099)	0.464 (0.6812)	0.337 (0.5805)

Based on this model, the direction of influence among the four indicators—anxiety, depression, psychological stress, and sleep—was adjusted to explore the interactions between the latent variables and to evaluate the significance and reasonableness of the data fitting.

**Fig 2:**
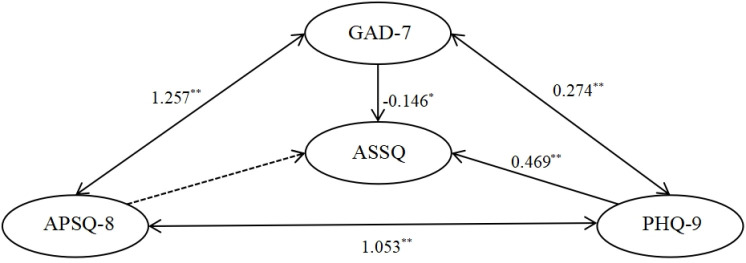
Modelling assumptions for interactions I: APSQ < -- > GAD-7 < -- > PHQ-9--- > ASSQ path analysis. (Note.X2 = 538.955, df = 199, p = 0.000, X2/df = 2.709, SRMR = 0.0477, RMSEA = 0.058, CFI = 0.943, GFI = 0.918).

**Fig 3:**
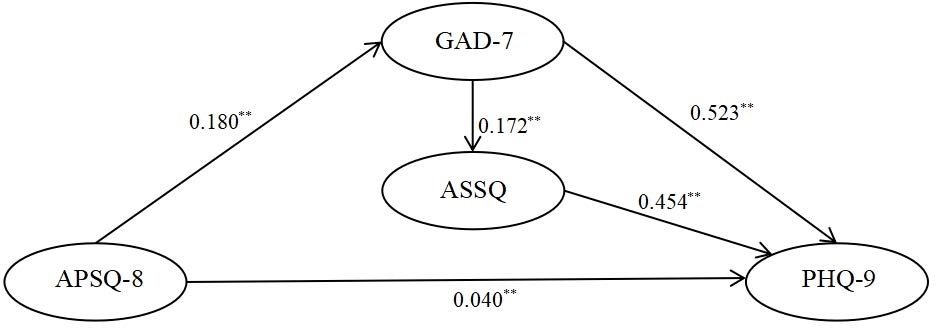
Modelling assumptions for the interactions II APSQ- > ASSQ- > PHQ-9, APSQ- > PHQ-9, APSQ- > GAD-7- > ASSQ- > PHQ-9, APSQ- > GAD-7- > PHQ-9 Path Analysis. (Note.X2 = 536.197, df = 199, p = 0.000, X2/df = 2.694, SRMR = 0.0480, RMSEA = 0.058, CFI = 0.943, GFI = 0.919).

**Fig 4:**
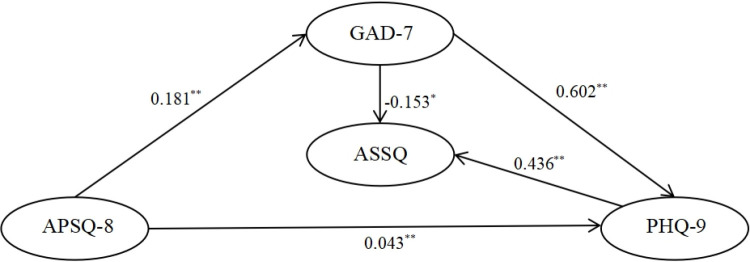
Model hypotheses for interactions III APSQ- > GAD-7- > ASSQ, APSQ- > ASSQ, APSQ- > PHQ-9- > ASSQ, APSQ- > GAD-7- > PHQ-9- > ASSQ Path analyses. (Note.X2 = 529.664, df = 198, p = 0.000, X2/df = 2.675, SRMR = 0.0473, RMSEA = 0.058, CFI = 0.944, GFI = 0.920).

The model assumptions and fitting results for the interaction relationships are illustrated in Figs 2–4. Each of the three relationship models, upon fitting, demonstrates the following: (1) APSQ-8 exhibits the weakest and non-significant effect on ASSQ; (2) APSQ has a positive and significant effect on both GAD-7 and PHQ-9; (3) GAD-7 positively and significantly influences PHQ-9; (4) Both GAD-7 and PHQ-9 significantly impact ASSQ.

Regarding the direction of influence, the structural effects and logical relationships presented in Model 2 (Fig 3) appear more coherent. Specifically, GAD-7 exerts a significant positive influence on ASSQ, which in turn significantly influences PHQ-9. For Model 2 (Fig 3), Table 4 provides the standardized coefficients and critical ratios for the five influence paths between APSQ, GAD-7, PHQ-9, and ASSQ, while Table 5 details the direct and indirect effects of APSQ on ASSQ.

**Table 4:** Summary of path coefficients.

Trails	Standardized coefficient	Residual Variance	Standard Error	Critical Ratio	*p*
APSQ --- > GAD-7	0.784	0.180	0.013	13.468	***
APSQ --- > PHQ-9	0.214	0.040	0.011	3.603	***
GAD-7--- > ASSQ	0.459	0.172	0.041	4.231	***
GAD-7--- > PHQ-9	0.643	0.523	0.057	9.142	***
ASSQ --- > PHQ-9	0.209	0.454	0.120	3.778	***

**Table 5:** Summary of mediating effects.

Path	Standardized Coefficient	Standard Error	*p*	95% Confidence Level	
Lower Limit	Upper Limit
Indirect path starting with APSQ					
APSQ--- > GAD-7--- > PHQ-9 APSQ--- > GAD-7--- > ASSQ--- > PHQ-9	0.580	0.056	0.000**	0.480	0.704
Direct path APSQ--- > PHQ-9	0.214	0.074	0.006**	0.055	0.347
Total effect	0.794	0.034	0.001**	0.716	0.855
Percentage of total indirect effect	73.0%				
Indirect path starting with GAD-7					
Indirect path GAD-7--- > ASSQ--- > PHQ-9	0.096	0.022	0.001**	0.054	0.142
Direct effect GAD-7--- > PHQ-9	0.643	0.068	0.001**	0.512	0.772
Total effect	0.740	0.066	0.001**	0.611	0.876
Percentage of total indirect effect	13.0%				

A reliability analysis of the influence pathways was conducted using the bootstrap method (n = 2000), resulting in 95% confidence intervals for each pathway. The analysis of direct and indirect effects within the model indicated that when the APSQ was used as the starting point, the direct effect coefficient of psychological stress (APSQ) on the PHQ-9 was 0.214. In contrast, the indirect effect coefficient of psychological stress on depression, mediated by anxiety and sleep disorders, was found to be 0.580, with the mediated effect of the indirect influence pathway accounting for 73.0% of the total mediated effect. When GAD-7 was considered as the starting point, the direct effect coefficient of GAD-7 on the PHQ-9 was 0.643, while the indirect effect coefficient of GAD-7 on depression, mediated by sleep disorders, was 0.096. The mediation effect of the indirect influence pathway accounted for 17.0%. Thus, while the direct effect of APSQ on PHQ-9 was significant, it represented only 26% of the total effect, whereas the indirect influence pathways of anxiety and sleep accounted for 73%. Additionally, sleep disorders contributed to 13.0% of the total influence of GAD-7 on PHQ-9.

## Discussion

This study investigated the relationship between sleep and three mental health indicators among Chinese high-level athletes, aiming to elucidate the interaction between sleep and mental health. The results confirmed a significant positive correlation among sleep, anxiety, depression, and perceived psychological stress. Additionally, anxiety and sleep disorders were found to partially mediate the relationship between perceived psychological stress and depression. The sequence observed in athletes indicated that perceived psychological stress occurs first, followed by the onset of anxiety and ultimately depression. Furthermore, sleep, as a vital health behavior for athletes, is significantly influenced by anxiety and plays a crucial role in the transition from anxiety to depression. However, in the present sample of athletes, sleep was not significantly affected by perceived psychological stress.

For high-level athletes, sleep is a crucial component of both psychological and physical recovery. It plays a significant role in the recovery of the immune and endocrine systems, as well as the nervous system, while also maintaining wakefulness and contributing to learning and memory functions. Collectively, these functions are essential for sports recovery and performance [45]. The sleep period represents the lowest point of metabolic activity during 24 hours, coinciding with high secretion levels of anabolic hormones, which create optimal conditions for physiological recovery [46]. Sleep is fundamental to the recovery, performance, and overall health of elite athletes [47,48]. However, sleep deprivation and poor sleep quality remain prevalent issues among this population [49]. On average, athletes obtain only 6.7 hours of sleep, which falls short of the optimal duration of 8.3 hours [50]. Elite athletes are particularly susceptible to sleep difficulties prior to major competitions, during intense training periods, and after prolonged travel [51,52]. Fatigue and anxiety stemming from inadequate sleep quality directly impact training and competition performance [53]. Furthermore, poor sleep quality leads to significant hormonal and cytokine alterations, characterized by reduced levels of testosterone and growth hormone, along with elevated cortisol levels, which may increase the risk of injury [54]. Additionally, poor sleep quality adversely affects mood and cognitive functions, including basic cognitive abilities such as memory, language, and visual processing, as well as executive functioning. These changes also influence endocrine functioning, cardiovascular health, immune responses, and mood state [55]. The neurobiological mechanisms linking sleep to mood and cognition involve neuronal activation in the sub-limbic cortex, lateral septum, and other brain regions [56].

Significant correlations have been established between sleep and perceived psychological stress, anxiety, and depression across various populations [57]. Research indicates that sleep serves a mediating role between anxiety and depression, with evidence suggesting that anxiety symptoms disrupt normal sleep patterns in college students, consequently increasing the risk of depression [58]. In this study, the mediating effect of sleep disorders on the relationship between anxiety and depression was found to be more pronounced in athletes compared to college students, highlighting the critical role of sleep in the mental health of this specific population. Anxiety was observed to have a more substantial impact on sleep, affecting total sleep time, sleep continuity, and sleep depth. Additionally, anxiety is closely linked to patterns of sleep disturbance, with decreased sleep continuity potentially representing a key pathological feature of anxiety [59]. Sleep disorders are also believed to be independently associated with depression. Notably, research indicates that sleep alterations in patients with major depression exhibit distinct characteristics compared to those with sleep disorders arising from other conditions. Furthermore, investigators have identified rapid eye movement sleep and alterations in the monoaminergic and neuroendocrine systems as a shared physiological basis underlying the interplay between sleep and depression [60].

The present study identified a pathway from perceived psychological stress to depression among high-level athletes, specifically through the sequence of perceived psychological stress, anxiety, sleep disturbances, and depression. Previous research has established perceived psychological stress as a precursor to anxiety and depression in athlete [61]. During the initial stages of stress perception, individuals strive to maintain elite professional performance. However, as coping strategies for stress begin to fail, anxiety emerges. If high performance is not adequately rewarded, individuals may eventually abandon these coping strategies [62]. This lack of reward contributes to the onset of anxiety, which subsequently progresses to depression. This study also introduces the observation of sleep disorders, building on prior research, and investigates the interplay between sleep and three mental health indicators. The findings indicate that high-level athletes, following the perception of stress and the resultant anxiety, often experience sleep disorders. These sleep disturbances, in conjunction with anxiety, can lead to the onset and progression of depression when not effectively managed. The identified pathway, beginning with stress perception, followed by anxiety and sleep issues, and culminating in depression, demonstrates a logical progression and offers a novel perspective for monitoring the mental health of athlete populations in the future.

Athletes are generally more adept at regulating their perception of stress and possess specific coping strategies [63]. However, long-term anxiety significantly impacts the quality of sleep among this population, which, in turn, exacerbates depressive symptoms. Therefore, close monitoring of mental health symptoms and sleep patterns should be integrated into daily operational practices. This study serves as an in-depth application of one of the IOC’s sport mental health assessment tools. Timely detection and intervention to prevent the deterioration of mental health indicators in athletes are crucial, as sleep disorders serve as a convenient and straightforward external monitoring indicator and play a vital role in the progression from anxiety to depression.

## Limitations and implications

This study has several limitations: (1) It employed a cross-sectional design, which restricts the ability to establish causal relationships between mental health indicators and sleep. A longitudinal study would be more effective in capturing time-related changes; (2) The sample consisted solely of Chinese athletes, which may limit the applicability of the findings to athletes from diverse cultural backgrounds or environments. Additionally, the focus on specific sports may not accurately represent athletes from other disciplines; (3) The study design did not account for factors that could influence the relationship between mental health and sleep, such as social support [64], training volume [65], stress coping strategies[66], and individual body perception [67]. Future research should incorporate these factors to enhance the comprehensiveness of the study.

## Conclusions

This study aimed to investigate the relationships and pathways of influence among anxiety, depression, psychological stress, and sleep disorders in Chinese high-level competitive athletes. The findings indicated that Chinese athletes exhibited similarities to previous studies regarding anxiety, depression, psychological stress, and sleep disorders, while also revealing distinct characteristics. Significant correlations were identified among anxiety, depression, psychological stress, and sleep disorders. Structural equation analyses indicated that psychological stress was positioned at the highest level, with anxiety and sleep disorders in the middle, and depression at the lowest level. Furthermore, anxiety and sleep disorders functioned as partial mediators between psychological stress and depression.

To address the mental health issues faced by competitive athletes, it is essential to enhance the application of the IOC Athlete Mental Health Screening Tool in future practices [40]. Health-related behaviors, such as sleep and diet, should be incorporated into the daily monitoring of athletes. Sports teams are encouraged to provide athletes with an optimal service model [68] that includes education, diagnosis, screening, and treatment. Additionally, athletes who meet the criteria for clinical interventions concerning mental health indicators should have access to an appropriate exit mechanism and corresponding medication regimen [69], or alternative therapies tailored to the sports environment. For individual athletes, there is a need for increased mental health awareness, social support [70], and effective stress coping strategies [71].

## Supporting Information

S1 Table Detailed information of the participants.
(DOCX)

S2 Text Health Questionnaire for High Performance Athletes.
(DOCX)
