# Supplementary material for: The relationship between sleep disorder and mental health in athletes and its mediating role: a cross-sectional study
Source: PLoS One. 2025 Mar 24;20(3):e0319813. doi: 10.1371/journal.pone.0319813 (PMC11932476; doi:10.1371/journal.pone.0319813)
Supplement: S1 Table — (DOCX) [file pone.0319813.s001.docx]

**S1 Table. Detailed information of the participants**

| Variable | Total | International Master Athlete | Master Athlete | Division I Athlete |
| --- | --- | --- | --- | --- |
|  | N（%） | N（%） | N（%） | N（%） |
| Gender |  |  |  |  |
| Male | 256(51.1) | 19(3.8) | 109(21.8) | 128(25.5) |
| Female | 245(48.9) | 24(4.8) | 92(18.4) | 129(25.7) |
| Age |  |  |  |  |
| 18 | 94(18.8) | 2(0.4) | 27(5.4) | 65(13.0) |
| 19 | 110(22) | 4(0.8) | 28(5.6) | 78(15.6) |
| 20 | 81(16.2) | 5(1.0) | 37(7.4) | 39(7.8) |
| 21 | 49(9.8) | 2(0.4) | 22(4.4) | 25(5.0) |
| 22 | 69(13.8) | 4(0.8) | 36(7.2) | 29(5.8) |
| 23 | 26(5.2) | 3(0.6) | 13(2.6) | 10(2.0) |
| 24 | 18(3.6) | 4(0.8) | 12(2.4) | 2(0.4) |
| 25 | 22(4.4) | 5(1.0) | 12(2.4) | 5(1.0) |
| 26 | 16(3.2) | 5(1.0) | 7(1.4) | 4(0.8) |
| 27 | 8(1.6) | 6(1.2) | 2(0.4) | / |
| 28 | 4(0.8) | 1(0.2) | 3(0.6) | / |
| 29 | 4(0.8) | 2(0.4) | 2(0.4) | / |
| Sport Discipline |  |  |  |  |
| Swimming | 78(15.6) | 3(0.6) | 32(6.4) | 43(8.6) |
| Track and Field | 70(14) | 10(2.0) | 24(4.8) | 36(7.2) |
| Gymnastics | 25(5.0) | 4(0.8) | 10(2.0) | 11(2.2) |
| Rowing | 11(2.2) |  | 1(0.2) | 10(2.0) |
| Table Tennis | 28(5.6) | 1(0.2) | 7(1.4) | 20(4.0) |
| Badminton | 8(1.6) | 1(0.2) | 3(0.6) | 4(0.8) |
| Basketball | 5(1.0) | / | 1(0.2) | 4(0.8) |
| Canoeing/Kayaking | 8(1.6) | / | 4(0.8) | 4(0.8) |
| Cycling | 10(2.0) | / | 5(1.0) | 5(1.0) |
| Boxing | 5(1.0) | 2(0.4) | 1(0.2) | 2(0.4) |
| Fencing | 10(2.0) | / | 1(0.2) | 9(1.8) |
| Modern Pentathlon | 17(3.4) | 1(0.2) | 10(2.0) | 6(1.2) |
| Judo | 1(0.2) | 1(0.2) |  |  |
| Tennis | 45(9.0) | 3(0.6) | 24(4.8) | 18(3.6) |
| Martial Arts | 17(3.4) | 1(0.2) | 8(1.6) | 8(1.6) |
| Acrobatics gymnastics | 33(6.6) | 4(0.8) | 20(4.0) | 9(1.8) |
| Taekwondo | 2(0.4) | 1(0.2) | 1(0.2) | / |
| Calisthenics | 2(0.4) | / | / | 2(0.4) |
| Trampoline | 18(3.6) | / | 6(1.2) | 12(2.4) |
| Rugby | 7(1.4) | / | 3(0.6) | 4(0.8) |
| Soccer | 30(6.0) | 4(0.8) | 12(2.4) | 14(2.8) |
| Dive | 71(14.2) | 7(1.4) | 28(5.6) | 36(7.2) |
