# Supplementary material for: The relationship between sleep disorder and mental health in athletes and its mediating role: a cross-sectional study
Source: PLoS One. 2025 Mar 24;20(3):e0319813. doi: 10.1371/journal.pone.0319813 (PMC11932476; doi:10.1371/journal.pone.0319813)
Supplement: S2 Text — (DOCX) [file pone.0319813.s002.docx]

**S2 Text. Health Questionnaire for High Performance Athletes**

Dear Athlete:

Hello! Welcome to the High Performance Athlete Health Questionnaire. This questionnaire can only be completed by me, not on my behalf, and will take approximately 15 minutes to complete. This questionnaire is designed to help you get to know yourself and is not intended to be used to assess your ability. There are no right or wrong answers to the questionnaire, so please answer based on what you know or feel about yourself. All information you provide in the questionnaire will only be used for research purposes. Your personal information will be kept absolutely confidential, so please feel free to fill it out. Your careful answers are very important to us. The information you provide can help more high level athletes in the future. Please answer all questions honestly and according to your true thoughts.

**I. Sleep Behaviour**

1. how many hours did you actually sleep at night in recent times? (This may differ from the number of hours you spent in bed.)

□ 5 to 6 hours □ 6 to 7 hours □ 7 to 8 hours □ 8 to 9 hours □ More than 9 hours

2. Are you satisfied/dissatisfied with the quality of your sleep?

□ Very satisfied □ Fairly satisfied □ Neither dissatisfied nor satisfied □ Somewhat dissatisfied □ Very dissatisfied

3. In recent times, how long does it usually take you to fall asleep each night?

□ 15 minutes or less □ 16-30 minutes □ 31-60 minutes □ More than 60 minutes

4. How many days a week do you have trouble falling asleep?

□No □One to two times □Three to four times □Five to seven times

5. How often do you take sleeping aids (prescription or over-the-counter) per week in recent times?

□ No □ One to two times □ Three to four times □ Five to seven times

**II. Psychological**

*How often have you been bothered by the following in the last 4 weeks?*

1. I have a hard time getting along with my teammates

□1 never □2 rarely □3 sometimes □4 often □5 always

2. I find it hard to get myself to do the things I need to do

□1 never □2 rarely □3 sometimes □4 often □5 always

3. I feel less motivated

□1 Never □2 Rarely □3 Sometimes □4 Often □5 Always

4. I am irritable, angry or aggressive

□1 never □2 rarely □3 sometimes □4 often □5 always

5. I worry about injuries or my performance

□1 never □2 rarely □3 sometimes □4 often □5 always

6. I feel more pressure to train.

□1 never □2 rarely □3 sometimes □4 often □5 always

7. I find it difficult to cope with the pressure of selection

□1 never □2 rarely □3 sometimes □4 often □5 always

8. I am worried about my life after I retire as an athlete.

□1 never □2 rarely □3 sometimes □4 often □5 always

9. I need to drink alcohol or take some drugs to relax

□1 Never □2 Rarely □3 Sometimes □4 Often □5 Always

10. I engage in special risk-taking behaviour in the presence of others.

□1 never □2 rarely □3 sometimes □4 often □5 always

*In the past 2 weeks, how often have you been bothered by the following?*

11. Feeling nervous or anxious

□1 Never □2 A few days □3 More than half the time □4 Almost every day

12. Unable to stop or control worrying

□1 Never □2 Some days □3 More than half the time □4 Almost every day

13. Worry too much about different things

□1 never □2 some days □3 more than half the time □4 almost every day

14. Difficult to relax

□1 never □2 a few days □3 more than half the time □4 almost every day

15. Restlessness and difficulty in sitting still

□1 never □2 some days □3 more than half the time □4 almost every day

16. Become easily angry or irritable

□1 never □2 some days □3 more than half the time □4 almost every day

17. Feeling afraid, as if something terrible will happen

□1 never □2 some days □3 more than half the time □4 almost every day

*In the past 2 weeks, how often were you bothered by the following problems?*

18. No interest or pleasure in doing things

□1 Never □2 Some days □3 More than half the time □4 Almost every day

19. Feeling low, depressed or hopeless

□1 Never □2 Some days □3 More than half the time □4 Almost every day

20. Difficulty sleeping, unable to sleep, or sleeps too much

□1 never □2 some days □3 more than half the time □4 almost every day

21. Feeling tired or having low energy

□1 Never □2 Some days □3 More than half the time □4 Almost every day

22. Loss of appetite or overeating

□1 Never □2 Some days □3 More than half the time □4 Almost every day

23. Feeling bad about yourself - feeling like a failure or feeling like you have let yourself or your family down

□1 never □2 some days □3 more than half the time □4 almost every day

24 Difficulty concentrating, e.g. reading the newspaper or watching TV

□1 never □2 some days □3 more than half the time □4 almost every day

25. Moves or talks so slowly that others may notice. Or the opposite: irritable to the point of moving more than usual

□1 never □2 some days □3 more than half the time □4 almost every day

26. thinks it is better to die or hurt himself in some way

□1 never □2 a few days □3 more than half the time □4 almost every day
